# The prognostic value of COL3A1/FBN1/COL5A2/SPARC-mir-29a-3p-H19 associated ceRNA network in Gastric Cancer through bioinformatic exploration

**DOI:** 10.7150/jca.45378

**Published:** 2020-06-16

**Authors:** Hongyu Shen, Lin Wang, Qinnan Chen, Juqing Xu, Jin Zhang, Leping Fang, Jun Wang, Weifei Fan

**Affiliations:** 1 Department of Hematology and Oncology, Department of Geriatric Lung Cancer Laboratory, Geriatric Hospital of Nanjing Medical University, Jiangsu Province Geriatric Hospital, Nanjing, Jiangsu, China.; 2 Department of Clinical Medicine, Jiangsu Health Vocational College, Nanjing, Jiangsu, China.; 3 Department of General Practice, Geriatric Hospital of Nanjing Medical University, Jiangsu Province Geriatric Hospital, Nanjing, Jiangsu, China.

**Keywords:** gastric cancer, bioinformatic analysis, competing endogenous RNA (ceRNA), prognosis, long noncoding RNA (lncRNA), microRNA (miRNA)

## Abstract

Increasing studies on malignant tumors have proposed a new competing endogenous RNA (ceRNA) regulatory mechanism that mRNA, miRNA and lncRNA interact with each other. However, the mRNA-miRNA-lncRNA associated ceRNA network in gastric cancer remains unknown. We used online bioinformatic softwares to predict the hub genes and their upstream miRNAs and lncRNAs in gastric cancer, and then performed survival analyses. After collecting gastric cancer tissue samples and performing PCR experiments, the correlations among predicted mRNA, miRNA and lncRNA were further verified. A total of 101 up-regulated significant differentially expressed genes (DEGs) and 219 down-regulated significant DEGs in gastric cancer were confirmed. Functional enrichment analyses of these significant DEGs indicated that they were potentially enriched in some pathways involved in tumor malignant biological processes or metabolism. Then, we identified 20 hub genes in the PPI networks. Combined with expression and survival analyses, 8 up-regulated genes and 1 down-regulated gene were identified as central genes and acted as important prognostic roles in gastric cancer. 17 miRNAs were confirmed that might potentially regulate the expressions of these central genes. But only 8 out of them indicated better outcome in gastric cancer. Further, 79 lncRNAs were predicted that might have the potence to combine with the 8 central miRNAs. The lncRNA H19 was eventually defined as a central lncRNA by survival analyses. Stimultaneously, we found that there were certain interactions among lncRNA, miRNA and mRNAs in 50 gastric cancer tissues by qRT-PCR. Moreover, the high expression of H19 is associated with advanced TNM stage, primary tumor and lymph nodes, indicating a poor prognosis. In summary, we uncovered the prognostic value of COL3A1/FBN1/COL5A2/SPARC-mir-29a-3p-H19 ceRNA network in gastric cancer.

## Introduction

Gastric cancer (GC) remains the fifth most frequently malignancy and the third leading cause of cancer death worldwide especially in East Asia, and ranks as a growing health-threatening disease, causing over 1,000,000 new cases and approximately 783,000 deaths in 2018 worldwide 1. Due to the lack of typical early symptoms, the majority of GC patients are diagnosed at advanced stages accompanied with malignant proliferation, extensive invasion and distant metastasis, with a median survival of only 3-5 months 2, 3. Despite great efforts such as surgery and adjuvant chemotherapy have been offered, the prognosis of GC patients has only slightly improved 4. To date, however, there has been little exploration on promising molecular biomarkers that could explain the risk of cancer recurrence and metastasis. Therefore, a comprehensive understanding of the mechanisms involved in the development of GC is urgently needed for improving the diagnosis, treatment strategy and prognosis of this disease.

Recently, long noncoding RNAs (lncRNAs) have received great attention in elucidating the complex mechanisms in malignant biological processes such as carcinogenesis, recurrence, metastases and drug resistance 5. Increasing studies proposed a new regulatory mechanism in which lncRNAs may act as competing endogenous RNAs (ceRNAs) and engage in crosstalk with mRNAs by using miRNA response elements (MREs) to competitively sponge their miRNAs 6-8. Intriguingly, more and more scholars and researchers are beginning to conduct a seris of researches on lncRNA-miRNA-mRNA ceRNA network in their respective fields, including cancer. For instance, FOXP4-AS1 post-transcriptionally regulated FOXP4 by acting as ceRNA to sponge to miR-3184-5p in prostate cancer 9, while FBXL19-AS1 influenced breat cancer cells progression via a ceRNA-mediated regulatory network to bind to miR-718 10. Other studies have investigated that ZEB1-AS1 can promote malignant behaviors of cancer cells through miRNA-mediated mechanisms 11-13. However, our current understanding on lncRNA-miRNA-mRNA is dificient.

In our study, firstly we obtained differentially expressed genes (DEGs) by screening four GEO datasets. Then, pathway enrichment analyses of these differentially expressed genes were performed. Besides, we also conducted protein-protein interaction (PPI) analyses and identified the hub genes. Considering the expression and survival analyses of hub genes in GC, 8 up-regulated genes and 1 down-regulated gene were selected for subsequent study. Immediately after, upstream miRNAs and lncRNAs were predicted based on online tools. In addition, we further assessed the prognostic values of these miRNAs and lncRNAs in GC. The correlation analyses of mRNAs, miRNAs and lncRNAs were also accomplished. Eventually, a split-new ceRNA regulatory network related to the prognosis of patients with GC was successfully inaugurated. Enthusiastically, based on our current analysis, all RNAs in the ceRNA network can be used to indicate the prognostic role of GC. In the future, they may also be promising as novel treatment strategies for GC.

## Material and Methods

### Data collection

The microarray datasets that compared gene expression between GC tissues and nontumorous/normal tissues were downloaded on the Gene Expression Omnibus database (http://www.ncbi.nlm.nih.gov/geo/). Only datasets containing more than 10 cancer/normal samples were included. We finally chose four datasets (GSE13911, GSE79973, GSE19826 and GSE54129) through screening the titles and abstracts of these datasets. The datasets only provided gene expression data instead of other non-coding RNA expression data. Based on Affymetrix Human Genome U133 Plus 2.0 Array (GPL570) platform, GSE13911 dataset was submitted from Italy group and other three datasets were all from China groups.

### Differential gene expression analysis

We employed the differential gene expression analyses on an online analytic tool GEO2R (https://www.ncbi.nlm.nih.gov/geo/geo2r) from the GEO database to retrieve DEGs among the four datasets. DEGs were considered as significantly different if they meet the conditions of |log_2_FC|>1 and p-value<0.05. In addition, VENNY 2.1.0 (http://bioinfogp.cnb.csic.es/tools/venny/index.html) was performed to draw the Venn diagrams. The DEGs that were concentrated in all four datasets were considered as the significant DEGs, including up-regulated significant DEGs and down-regulated significant DEGs.

### Gene ontology and KEGG pathway enrichment analysis

Gene Ontology (GO) functional analysis and Kyoto Encyclopedia of Genes and Genomes (KEGG) pathway enrichment analysis were submitted to ClueGO+CluePedia in Cytoscape 3.6.1. The GO classification includes biological process (GO-BP), cell component (GO-CC), and molecular function (GO-MF). We chose the BP for further exploration. For GO-BP analysis, the number of enriched genes was required to be greater than or equal to 10 and the proportion of genes was greater than 4%. For both GO and KEGG pathway, p-value < 0.05 was considered as statistically significant.

### Establishment of PPI networks and identification of hub genes

Based on the DEGs identified, the Search Tool for the Retrieval of Interacting Genes (STRING) database (http://stringdb.org/) was adopted to construct the PPI networks 14. Interactors with combined confidence score ≥ 0.4 were selected and high-resolution bitmaps were downloaded from the webpage. According to the degree of connectivity, the CytoHubba app was used to screen modules of hub genes in the PPI networks 15. Based on node degree, the top 30 hub genes were visualized using the Cytoscape 3.6.1 software.

### Gene expression analysis

In The Cancer Genome Atlas Program (TCGA) project, there are only 36 normal gastric samples, and the sample size is insufficient to compare the gastric cancer with normal controls. Gene Expression Profiling Interactive Analysis (GEPIA) (http://gepia.cancer-pku.cn/about.html) is a newly developed interactive web server for analyzing the RNA sequencing expression data of 9736 tumors and 8587 normal samples from the TCGA and the GTEx projects, using a standard processing pipeline 16. GEPIA2 is an updated version 17. In our study, the expression levels of central genes and lncRNAs in GC was analyzed by GEPIA2 database, which contains 408 gastric cancer samples and 211 normal controls. Besides, the |log_2_FC| threshold was set to be 1 and p-value was less than 0.05.

### Survival analysis

Prognostic values of genes in GC were analyzed using Kaplan-Meier plotter database, which is capable to assess the effect of 54k genes on survival in 21 cancer types 18. The mRNA RNA-seq data of GC were obtained from “Pan-cancer” item in Kaplan-Meier plotter database. After entering these genes into the database, the hazard ratio (HR) with 95% confidence interval (CI) and logrank p-value were automatically calculated. Survival curves were also directly shown on the webpage. Logrank p-value < 0.01 was used to narrow the range of DEGs. The miRNA subsystems include 11k samples from 20 different cancer types and miRNA survival data were acquired using the similar method. Logrank p-value < 0.05 was recognized as statistically significant. Prognostic values of lncRNAs in GC were performed through UALCAN (http://ualcan.path.uab.edu/index.html), which is a comprehensive, user-friendly, and interactive web resource for analyzing cancer OMICS data 19. UALCAN is designed to provide patient survival information based on gene expression including lncRNAs and p-value < 0.05 was regarded as statistically significant.

### Prediction of miRNA

In our study, miRTarbase (http://mirtarbase.mbc.nctu.edu.tw/php/search.php), an experimentally validated microRNA-target gene interactions database, was used to predict the upstream miRNAs of hub genes 20. As a database, miRTarBase has collected more than three hundred and sixty thousand miRNA-target interactions (MTIs). Generally, the accumulated MTIs were validated experimentally by reporter assay, western blot, microarray and next-generation sequencing experiments. In order to obtain more precise results, only MTIs that were validated by reporter assay included. Prognostic values of these predicted miRNAs were further evaluated by Kaplan-Meier plotter database as described above.

### Prediction of lncRNA

Upstream lncRNAs of miRNAs were predicted using miRNet database (https://www.mirnet.ca/), which is a user-friendly, integrated tool suite designed for comprehensive analysis and functional interpretation of miRNAs and xeno-miRNAs 21, 22. 'Organism-H.sapiens (human)', 'ID type-miRBase ID', 'Tissue-Gastric' and 'Target type-lncRNAs' were set as the selection criteria.

### Correlation analysis

StarBase database (http://starbase.sysu.edu.cn/index.php) is an open-source platform for studying the miRNA-ncRNA, miRNA-mRNA, ncRNA-RNA, RNA-RNA, RBP-ncRNA and RBP-mRNA interactions from CLIP-seq, degradome-seq and RNA-RNA interactome data 23, 24. On the basis of this platform, the correlation analyses in our study were conducted and p-value < 0.05 was considered as statistically significant.

### Tissue samples and clinical data collection

A total of 50 tumor tissues and adjacent normal tissues were collected from GC patients who underwent surgery at Geriatric Hospital of Nanjing Medical University. According to the 8th edition of the American Joint Committee on Cancer (AJCC) TNM Staging System for GC and histopathological evaluation, all patients were confirmed. Clinicopathological characteristics of GC patients are shown in Table 3. No neoadjuvant therapy was performed on these patients before surgery. Prior to real-time quantitative PCR (RT-qPCR) analysis, all tissues were stored at -80°C. All human tissue samples were obtained with written informed consent from all subjects, and this project was approved by the Research Ethics Committee of Geriatric Hospital of Nanjing Medical University.

### RT-qPCR Assay

Total RNA was isolated using TRIzol reagent (Invitrogen) from tissues. Total RNA (1 μg) was reverse transcribed by using BuSuperScript RT Kit (Biouniquer Technology, Nanjing, China) following the manufacturer's instruction. For detection of lncRNA, miRNA and mRNA expression levels, RT-qPCR was then performed on LightCycler®480 (Roche, Switzerland) with SYBR Green PCR Master Mix (Roche, Australia). Before calculation using the ΔΔCt method, the levels of GAPDH were used to normalize the relative expression levels of lncRNA and mRNA, and the levels of small nuclear U6 were used to normalize the miRNA expression levels. The primers are provided in Table S5.

### Statistical analysis

Statistical analysis was performed using SPSS 21.0 statistical package. All experiments were carried out at least three times independently. The relationships between H19 expression and clinicopathologic parameters were analyzed by chi-square test and the Mann-Whitney U test. The Kaplan-Meier method was employed to demonstrate the relationship between H19 expression and the prognosis of GC patients. Statistical comparisons among expressions of lncRNA, miRNA and mRNAs were undertaken using the Spearman correlation. P < 0.05 was considered statistically significant. Graphs were generated on GraphPad.

## Results

### Screening for significant DEGs in gastric cancer

**Figure 1:**
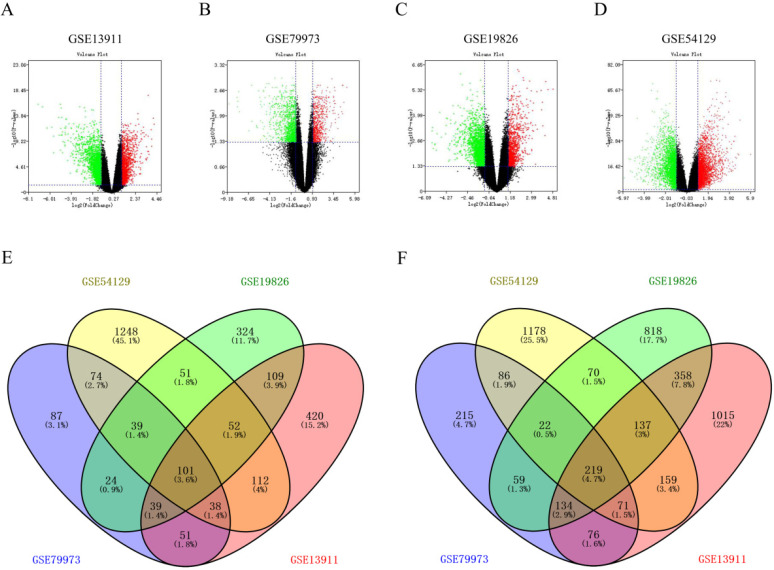
** Screening for significant DEGs in gastric cancer. (A-D)** Volcano plots from four datasets (GSE13911, GSE79973, GSE19826 and GSE54129). X axis represents the p-value of the logarithmic transformation, and Y axis represents the average gene expression differences between gastric cancer samples and normal samples. The four volcano plots showed all DEGs; red dots and green dots represent the up-regulated and down-regulated genes in gastric cancer samples, respectively; black dots represent genes that are not differentially expressed between gastric cancer samples and controls. |log_2_FC| >1 and p-value < 0.05 were set as the cut-off criteria. **(E-F)** Venn diagrams of up-regulated and down-regulated DEGs from four GSE datasets. The genes taking the intersection are considered to be significant DEGs.

Firstly, through screening gene expression microarrays related to GC from the GEO database, four datasets (GSE13911, GSE79973, GSE19826 and GSE54129) were ultimately selected. Secondly, differential gene expression analysis was performed by GEO2R, with the criteria of |log_2_FC| > 1 and p-value < 0.05. The DEGs in each dataset were separately displayed in Figure 1A, Figure 1B, Figure 1C and Figure 1D. Thirdly, we further searched some significant DEGs which were simultaneously appeared in the four datasets. As shown in Figure 1E and Figure 1F, a total of 101 up-regulated significant DEGs and 219 down-regulated significant DEGs in GC were confirmed. These up-regulated and down-regulated significant DEGs were completely listed in Table S1 and Table S2, respectively. Subsequently, these significant DEGs were subject to in-depth analysis.

### Function analysis of the significant DEGs

GO and KEGG pathway analyses were performed to further reveal the biological processes and relevant pathways of these significant DEGs (Table S6). ClueGO, a plugin of the Cytoscape software, was involved in subsequent analyses.

**Figure 2:**
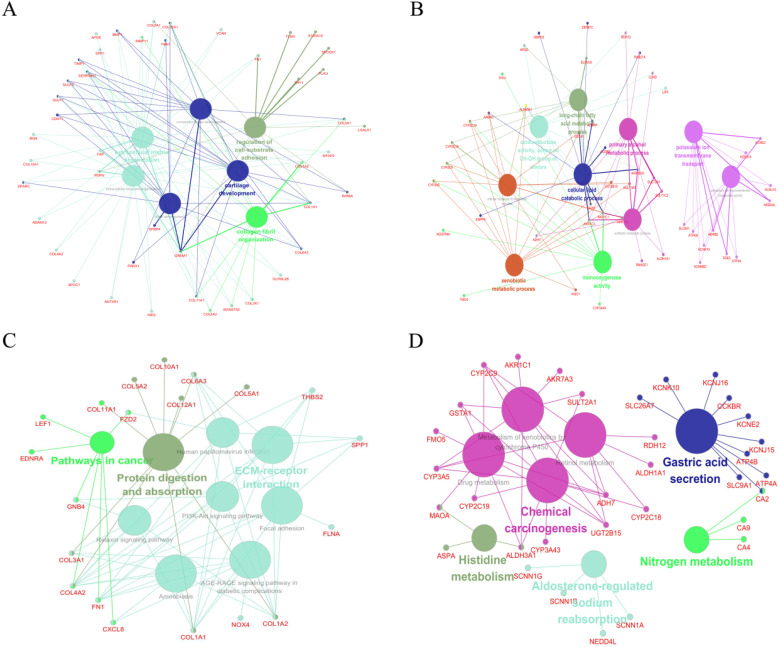
** Function analysis of the significant DEGs. (A)** The enriched GO biological processes (BP) of the up-regulated significant DEGs. **(B)** The enriched GOBP of the down-regulated significant DEGs. **(C)** The enriched KEGG pathways of the up-regulated significant DEGs. **(D)** The enriched KEGG pathways of the down-regulated significant DEGs.

For up-regulated significant DEGs, the enriched GO-BP functions included extracellular structure organization, extracellular matrix organization, collagen fibril organization, cartilage development, connective tissue development, regulation of cell-substrate adhesion and bone development (Figure 2A). Additionally, Figure 2C indicated that these up-regulated significant DEGs were significantly enriched in some cancer-associated pathways, such as protein digestion and absorption, pathways in cancer, PI3K-Akt signaling pathway, focal adhesion and ECM-receptor interaction.

As shown in Figure 2B, the enriched GO-BP functions for down-regulated significant DEGs included primary alcohol metabolic process, xenobiotic metabolic process, long-chain fatty acid metabolic process, oxidoreductase activity (acting on CH-OH group of donors), cellular response to xenobiotic stimulus, potassium ion transmembrane transport, antibiotic metabolic process, cellular lipid catabolic process, monooxygenase activity and potassium ion transmembrane transporter activity. Similarly, some enriched KEGG pathways were also discovered, among which gastric acid secretion, chemical carcinogenesis, drug metabolism and metabolism of xenobiotics by cytochrome P450 were the most highly enriched pathways (Figure 2D).

### Construction and deep analysis of PPI network

**Figure 3:**
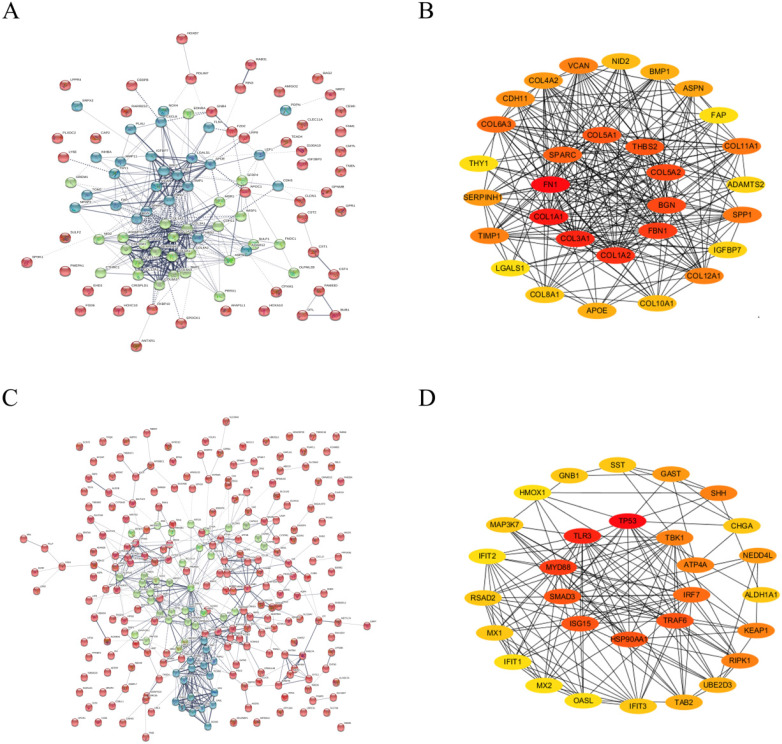
** The top 30 hub genes determined in PPI networks. (A)** The PPI networks of the up-regulated significant DEGs. **(B)** The top 30 hub genes of the up-regulated significant DEGs. **(C)** The PPI networks of down-regulated significant DEGs. **(D)** The top 30 hub genes of the down-regulated significant DEGs.

Based on STRING database analysis, PPI networks with the up-regulated significant DEGs and down-regulated significant DEGs were established, as shown in Figure 3A and Figure 3C, respectively. By calculating the node degree, some hub genes among these significant DEGs were determined. For a better visual experience, the interactions of top 30 up-regulated (Figure 3B) and down-regulated (Figure 3D) hub genes were reconstructed through Cytoscape software. Besides, the top 30 hub genes with relevant node degrees were listed in Table 1. The top 10 up-regulated hub genes were FN1, COL1A1, COL3A1, COL1A2, FBN1, BGN, COL5A2, THBS2, COL5A1 and SPARC, and top 10 down-regulated hub genes were TP53, TLR3, MYD88, SMAD3, ISG15, HSP90AA1, TRAF6, IRF7, ATP4A and TBK1. The 20 hub genes were selected for following analyses.

### Ascertainment of central genes in gastric cancer

**Figure 4:**
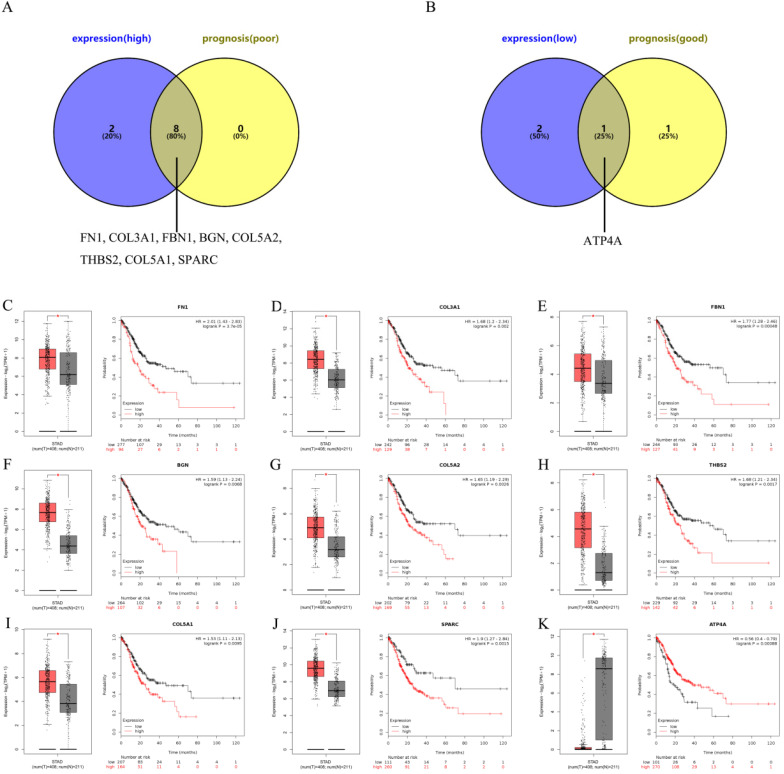
** Ascertainment of central genes in gastric cancer. (A-B)** Ascertainment of central genes among the top 10 hub genes of the significant up-regulated and down-regulated DEGs by integrating expression and prognosis analyses base on GEPIA and Kaplan Meier-plotter databases, respectively. **(C-K)** Expressions and prognostic values of the central genes (FN1, COL3A1, FBN1, BGN, COL5A2, THBS2, COL5A1, SPARC and ATP4A) in gastric cancer.

To further ascertain central genes in GC, we used the GEPIA and Kaplan-Meier plotter databases to determine the expression and prognosis of the top 10 up- and down-regulated central genes, respectively. Taken the results of expression analysis and survival analysis into consideration, 8 up-regulated hub genes (FN1, COL3A1, FBN1, BGN, COL5A2, THBS2, COL5A1 and SPARC) were discovered not only significantly up-regulated in gastric cancer groups but also predicted poor prognosis in patients with gastric cancer (Figure 4A and Figure 4C-J). Only 1 down-regulated hub gene (ATP4A) had a low expression and a good prognosis (Figure 4B and Figure 4K). In the following analyses, we are impassioned to further explore the 9 central genes, including 8 up-regulated hub genes and 1 down-regulated hub gene.

### Prediction and identification of upstream central miRNAs of central genes

**Figure 5:**
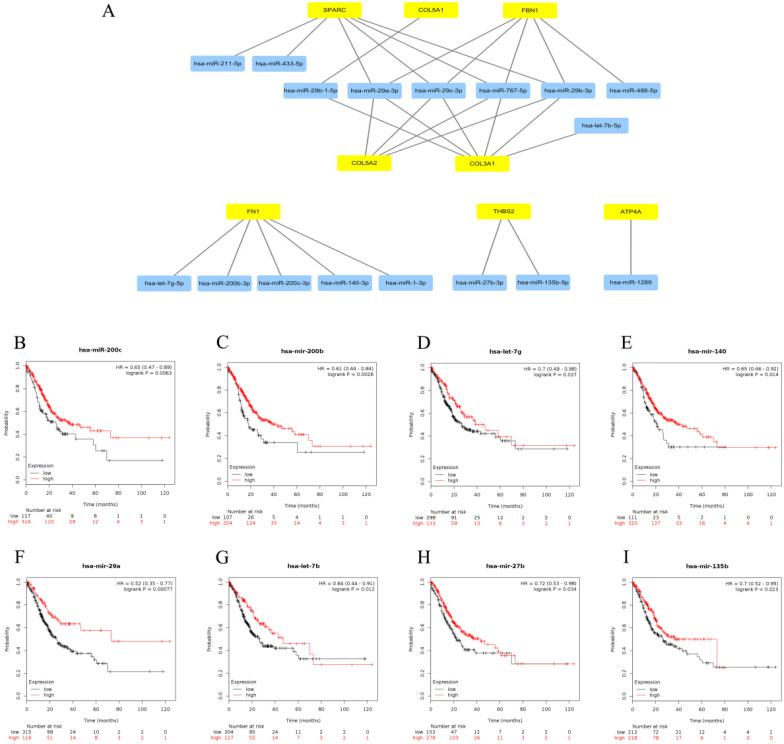
** Identification of upstream central miRNAs of central genes. (A)** Establishment of miRNA-gene network based on Cytoscape software. **(B-I)** Prognostic values of central miRNAs (miR-200c-3p, miR-200b-3p, let-7g-5p, miR-140-3p, miR-29a-3p, let-7b-5p, miR-27b-3p and miR-135b-5p) in gastric cancer.

Subsequently, upstream miRNAs of the 9 central genes were predicted relying on miRTarBase. As noted above, we only included microRNA-target gene interactions verified by reporter assay in our study. Eventually, a total of 17 miRNAs were confirmed that might potentially regulate the expression of 8 central genes (FN1, COL3A1, FBN1, COL5A2, THBS2, COL5A1, SPARC and ATP4A), as presented in Figure 5A and Table S3. No potential upstream miRNAs were observed for the BGN gene. Focusing on the recognized opposite relationship between target genes and miRNAs, we hypothesized that the upstream miRNAs of the 7 up-regulated central genes should theoretically show good prognosis, and the down-regulated gene ATP4A should act as an unfavorable prognostic role. Thence, we further explored the prognostic values of the 17 predicted miRNAs by Kaplan-Meier plotter database. Survival analysis displayed that 8 (miR-200c-3p, miR-200b-3p, let-7g-5p, miR-140-3p, miR-29a-3p, let-7b-5p, miR-27b-3p and miR-135b-5p) out of 17 miRNAs were considered as better outcome biomarkers in GC patients as shown in Figure 5B-I. We sensed the 8 miRNAs as the central miRNAs.

### Prediction and identification of upstream central lncRNAs of central miRNAs

**Figure 6:**
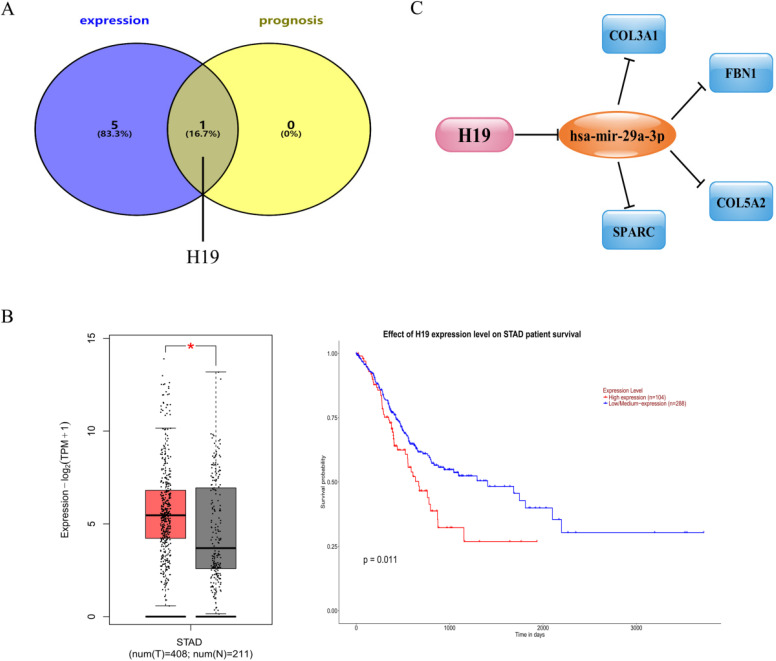
** Identification of upstream central lncRNAs of central miRNAs. (A)** Identification of central lncRNAs among the predicted lncRNAs by integrating expression and prognosis analyses using GEPIA and UALCAN databases, respectively. **(B)** Expression and prognostic value of H19 in gastric cancer. **(C)** The map of the novel mRNA-miRNA-lncRNA ceRNA network associated with prognosis of gastric cancer.

It is well known that lncRNA can interact with miRNA as a competitive endogenous RNA (ceRNA) to regulate the expression of target genes, which play an important role in the occurrence and development of tumors 25, 26. In light of this theory, the lncRNAs that might have the potence to combine with the 8 central miRNAs (miR-200c-3p, miR-200b-3p, let-7g-5p, miR-140-3p, miR-29a-3p, let-7b-5p, miR-27b-3p and miR-135b-5p) were predicted by an online miRNet database. A total of 79 lncRNAs were confirmed as displayed in Table S4. Based on the classical ceRNA hypothesis, there is an inverse correlation between lncRNA and miRNA. Therefore, we used the GEPIA database to analyze the expression of these lncRNAs in GC. Only 6 (H19, HCP5, LINC00511, MALAT1, OIP5-AS1 and RP11-618G20.1) out of 79 lncRNAs were significantly up-regulated in gastric cancer tissues compared to normal controls. Subsequently, survival analysis on an online UALCAN database of the 6 up-regulated lncRNAs indicated that patients with high expression of H19 had an unfavorable prognosis. After comprehensive consideration, the lncRNA H19 was defined as a central lncRNA (Figure 6A-B).

### Establishment of mRNA-miRNA-lncRNA network in gastric cancer

Through a series of online analyses of bioinformatics, we established an essential mRNA-miRNA-lncRNA competitive endogenous RNA network associated with prognosis in GC. The network contained a total of 9 pairs, including 4 mRNA-miRNA pairs (COL3A1-mir-29a-3p, FBN1-mir-29a-3p, COL5A2-mir-29a-3p and SPARC-mir-29a-3p), 4 mRNA-lncRNA pairs (COL3A1-H19, FBN1-H19, COL5A2-H19 and SPARC-H19) and only one miRNA-lncRNA pair (mir-29a-3p-H19). This network was drawn in Figure 6C.

As we all known, lncRNA can attenuate the inhibition of mRNA expression by competitively binding to miRNA. In view of this hypothesis, there are negative correlations between miRNAs and lncRNAs or mRNAs and positive relationships between mRNAs and lncRNAs. Furthermore, the gastric cancer associated TCGA data from StarBase database were introduced to profoundly determine the correlations of mRNA-miRNA, mRNA-lncRNA and miRNA-lncRNA pairs in the established network. As shown in Table 2, all pairs as described above were consistent with the ceRNA mechanism (p<0.05). Taken together, we established a brand new mRNA-miRNA-lncRNA network, COL3A1/FBN1/COL5A2/SPARC-mir-29a-3p-H19, which is correlated to the prognostic value of patients with GC. This network is also promising as a novel biomarker for diagnosis or a therapeutic strategy for GC.

### Preliminary validation of the mRNA-miRNA-lncRNA network in gastric cancer tissue samples

**Figure 7:**
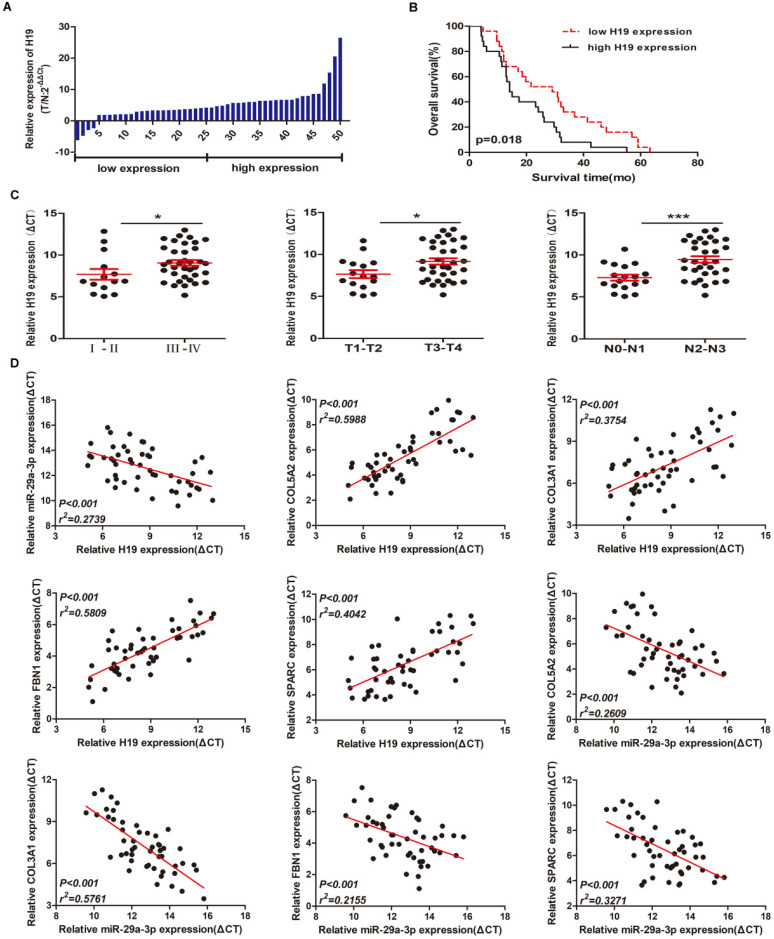
** Preliminary validation of the mRNA-miRNA-lncRNA network in gastric cancer tissue samples.** (A) H19 expression in GC tissues (n = 50) compared with normal tissues (n = 50) was examined by qRT-PCR and normalized to GAPDH expression. (B) Kaplan-Meier overall survival curves according to H19 expression levels. (C) The relationships between H19 expression and clinicopathological characteristics (TNM stage, primary tumor and lymph nodes). (D) Spearman correlation analysis among the expression of H19, miR-29a-3p and mRNAs (COL3A1/FBN1/COL5A2/SPARC).

Quantitative PCR experiments showed that lncRNA H19 expression was increased in 50 GC tissue samples compared with paired adjacent normal tissues (Figure 7A). To assess the significance of H19 overexpression in GC, we evaluated the correlation between H19 expression and patient clinicopathological characteristics. 50 GC patients were classified into two groups according to the median ratio (4.24) of relative H19 expression in tumor tissues: high H19 group (n=25, H19 expression ratio≥median ratio) and low H19 group (n=25, H19 expression ratio≤median ratio). As shown in Table 3, higher levels of H19 were significantly associated with advanced TNM stage (p=0.012), primary tumor (p=0.015), and lymph nodes (p=0.018). However, there was no obvious relationship between H19 expression and other clinical parameters such as age (p=0.569), gender (p=0.564), smoking history (p=0.771), drinking history (p=0.777) or differentiation (p=0.395). Kaplan-Meier survival analysis was used to examine the association between H19 expression and GC patient prognosis. Notably, patients with higher H19 expression levels had significantly shorter overall survival time than those expressing lower levels of H19 (p=0.018) (Figure 7B). We also find that patients with TNM stage III-IV, primary tumor T3-T4 and lymph nodes N2-N3 showed higher H19 expression than patients with TNM stage I-II, primary tumor T1-T2 and lymph nodes N0-N1 (Figure 7C). These results suggested that H19 might play an essential biological role in GC tumorigenesis and progression. Spearman correlation analysis showed that the expression between H19 and miR-29a-3p and the expression between miR-29a-3p and mRNAs (COL3A1/FBN1/COL5A2/SPARC) were inversely proportional, while the expression between H19 and mRNAs (COL3A1/FBN1/COL5A2/SPARC) was positively correlated. The p-value were all statistically significant (Figure 7D).

## Discussion

The incidence and mortality of gastric cancer have reduced substantially since great progress in surgery, chemotherapy and targeted therapy over the past 50 years. However, there are still a large number of gastric cancer patients suffered recurrence and metastasis after surgical resection, particularly those with diffuse-type in Asia. It's a great challenge for us to search for efficient biomarkers of prognosis 27. The biological function of miRNAs have been studied extensively in a variety of cancers over the past few decades, including GC 28, 29. At present, as important members in cell processes and human diseases, the newly discovered lncRNAs have attracted more and more researchers' attention 30. After the ceRNA hypothesis first proposed by Salmena et al. 6, more and more experiments have been conducted regarding ceRNAs in human cancers. For instance, lncRNA CASC11 was overexpressed in hepatocellular carcinoma and promotes cell proliferation by suppressing miRNA-188-5p 31; Bai et al. found that lncRNA LOXL1-AS1/miR-let-7a-5p/EGFR axis obviously changed proliferation, migration and apoptosis of drug-resistant DU-145 Cells, providing a promising treatment approach for drug-resistant prostate cancer patients 32; Similar studies have been reported in gastric cancer. Lu et al. indicated that lncRNA BC032469 acted as a novel ceRNA to modulate hTERT expression by sponging miR-1207-5p and promoted proliferation in gastric cancer 33; Li et al. found that MIAT competitively binded to miR-29a-3p and consequently upregulated the expression of HDAC4, highlighting the involvement of the MIAT/miR-29a-3p/HDAC4 axis in the cell biological behaviors of GC 34; LincHOTAIR epigenetically repressed miR34a by binding to PRC2 and contributed to the epithelial-to-mesenchymal transition (EMT), finally accelerating metastasis in human gastric cancer 35. However, there's a shortage in comprehensive analyses of ceRNAs and mRNAs in gastric cancer. Herein, it aims to study a specific prognosis-related ceRNA network in gastric cancer via the “mRNA-miRNA-lncRNA” model rather than the “lncRNA miRNA-mRNA” model. Encouragingly, we have established a new mRNA-miRNA-lncRNA network in which each RNA play a significant prognostic role in gastric cancer.

In our current study, we have totally selected 320 significant DEGs including 101 up-regulated and 219 down-regulated DEGs through extraction from four GEO datasets. GO and KEGG are widely used as functional enrichment analysis for numerous genes 36, 37. The results of these significant DEGs indicated that they were potentially enriched in some pathways involved in tumor malignant biological processes or metabolism, including cell-substrate adhesion, PI3K-Akt signaling pathway, focal adhesion and ECM-receptor interaction, antibiotic metabolic process, chemical carcinogenesis, drug metabolism and so on. Plenty studies have shown that focal adhesion and cell adhesion own in dispensable positions in cancer invasion and metastasis, consequently leading to cancer progression 38, 39. In addition, the PI3K-Akt pathway has been considered as one of the essential pathways for tumor growth, invasion and even drug resistance 40. Thus, these significant DEGs may participate in the regulation of malignant biological behaviors of gastric cancer.

In order to systematically analyze the interactions and functions of these significant DEGs in gastric cancer, we obtained PPI networks on STRING database. As we all know, the more node degrees present in the PPI network, the greater the role that genes will play. Thus, we identified 20 hub genes in the two PPI networks according to the degree of node. To further determine central genes in gastric cancer, the top ten up- and down-regulated central genes were conducted by expression and survival analyses. The results showed that 8 up-regulated genes (FN1, COL3A1, FBN1, BGN, COL5A2, THBS2, COL5A1 and SPARC) and 1 down-regulated gene (ATP4A) may be the central genes in gastric cancer. Further searching for literatures, most of these central genes have been well investigated in gastric cancer. Before, it has been verified low expression of gastric cancer-associated fibroblasts-derived SPARC could lead to cancer stem cell transformation and 5-FU resistance 41; FN1 was significantly up-regulated in GC tissues and down-regulation of FN1 could inhibit the proliferation, migration and invasion of GC cells 42; Patients with low expression of THBS2 had a positive prognosis 43. These findings may support the accuracy of our bioinformatics analysis to a certain extent.

As mentioned above, miRNAs and lncRNAs can participate in the regulation of gene expression through the ceRNA mechanism. The upstream miRNAs of the central genes were first predicted. Survival analysis showed that patients with higher expression of 8 miRNAs (miR-200c-3p, miR-200b-3p, let-7g-5p, miR-140-3p, miR-29a-3p, let-7b-5p, miR-27b-3p and miR-135b-5p) had better overall survival in gastric cancer. Some of the 8 miRNAs have been reported for their tumor suppressive effects in gastric cancer. For example, miR-29a-3p showed a significant down-regulated expression in GC tumor tissues and high expression of miR-29a-3p inhibited cell proliferation and metastasis by targeting CDK2, CDK4, and CDK6 44; Tao et al. indicated that miR-27b-3p exerts tumor-suppressive effects in GC via the inhibition of oncogene ROR1 expression 45. Next, we further predicted 79 upstream lncRNAs of these central miRNAs. Based on TCGA data, integrating expression analysis and survival analysis of these lncRNAs, only 1 lncRNA (H19) was considered as the central lncRNA. Moreover, overexpression of lncRNA H19 has been determined to enhance the proliferation, invasion of gastric cancer and contributed to poor prognosis in GC patients in multiple studies 46-49. Naturally, a prognosis-related mRNA-miRNA-lncRNA ceRNA network in gastric cancer was successfully constructed. Correlation analysis of the RNA pairs in the established network further demonstrated that this network was absolutely compliant with the ceRNA hypothesis. Without any doubt, we have gained exciting discoveries through a series of bioinformatic analyses. Supplementally, we proved the ceRNA network through preliminary RT-qPCR validation in gastric cancer tissue samples; however, more experimental and clinical studies are needed in the future to fully elucidate this network, which will help to develop better strategies for the diagnosis and therapy of GC.

## Conclusion

In conclusion, we have established a novel mRNA-miRNA-lncRNA ceRNA network through integrating bioinformatic analysis and experiments on clinical samples. This network is significantly associated with the prognosis of patients with GC. In addition, it may also provide some new directions for studying the molecular mechanism of GC. However, further researches are still needed to validate this network.

## Supplementary Material

Supplementary tables.

## Figures and Tables

**Table 1:** The top 30 hub genes in PPI networks

Up-regulated genes		Down-regulated genes	
Name	Degree	Name	Degree
FN1	40	TP53	42
COL1A1	37	TLR3	22
COL3A1	36	MYD88	20
COL1A2	34	SMAD3	19
FBN1	29	ISG15	19
BGN	28	HSP90AA1	19
COL5A2	28	TRAF6	19
THBS2	25	IRF7	18
COL5A1	25	ATP4A	16
SPARC	24	TBK1	16
COL6A3	22	KEAP1	16
COL11A1	21	SHH	16
SPP1	21	RIPK1	16
COL12A1	20	GAST	15
TIMP1	20	NEDD4L	15
VCAN	20	UBE2D3	14
CDH11	19	TAB2	14
SERPINH1	18	CHGA	13
COL4A2	18	ALDH1A1	13
ASPN	15	GNB1	13
BMP1	14	SST	13
APOE	14	RSAD2	13
NID2	13	MX1	13
COL10A1	13	IFIT3	13
COL8A1	12	MAP3K7	13
IGFBP7	11	NQO1	12
ADAMTS2	11	HMOX1	12
FAP	10	MX2	12
THY1	10	IFIT2	12
LGALS1	10	OASL	12

**Table 2:** The correlations of mRNA-miRNA, mRNA-lncRNA and miRNA-lncRNA pairs in the established network by starBase database

lncRNA	miRNA	R	p-value
H19	hsa-mir-29a-3p	-0.241	2.52E-06
**miRNA**	**mRNA**	**R**	**p-value**
hsa-mir-29a-3p	COL3A1	-0.225	1.22E-05
hsa-mir-29a-3p	FBN1	-0.217	2.53E-05
hsa-mir-29a-3p	COL5A2	-0.246	1.60E-06
hsa-mir-29a-3p	SPARC	-0.214	3.10E-05
**lncRNA**	**mRNA**	**R**	**p-value**
H19	COL3A1	0.308	1.11E-09
H19	FBN1	0.196	1.29E-04
H19	COL5A2	0.289	1.13E-08
H19	SPARC	0.308	1.18E-09

**Table 3:** Correlation between expression of lncRNA H19 and clinicopathological characteristics of patients with gastric cancer

Characteristics	Cases (n)	H19		p value
		High (n)	Low (n)	
**Age (years)**				
≤60	22	10	12	0.569
>60	28	15	13	
**Gender**				
Male	20	11	9	0.564
Female	30	14	16	
**Smoking history**				
Yes	19	9	10	0.771
No	31	16	15	
**Drinking history**				
Yes	25	12	13	0.777
No	25	13	12	
**TNM stage**				
Ⅰ-Ⅱ	14	3	11	0.012^*^
Ⅲ-Ⅳ	36	22	14	
**Primary tumor**				
T1-T2	16	4	12	0.015^*^
T3-T4	34	21	13	
**Lymph nodes**				
N0-N1	18	5	13	0.018^*^
N2-N3	32	20	12	
**Differentiation**				
Poor	23	13	10	0.395
High/moderate	27	12	15	

## Abbreviations

ceRNA: competing endogenous RNA
lncRNA: long noncoding RNA
miRNA: microRNA
DEGs: differentially expressed genes
PPI: Protein-protein interaction
GC: Gastric cancer
MREs: miRNA response elements
GO: Gene Ontology
KEGG: Kyoto Encyclopedia of Genes and Genomes
BP: biological process
CC: cell component
MF: molecular function
STRING: the Search Tool for the Retrieval of Interacting Genes
GEPIA: Gene Expression Profiling Interactive Analysis
HR: hazard ratio
MTIs: miRNA-target interactions
EMT: epithelial-to-mesenchymal transition
TCGA: The Cancer Genome Atlas Program
AJCC: American Joint Committee on Cancer
