# Supplementary material for: The prognostic value of COL3A1/FBN1/COL5A2/SPARC-mir-29a-3p-H19 associated ceRNA network in Gastric Cancer through bioinformatic exploration
Source: J Cancer. 2020 Jun 16;11(17):4933–46. doi: 10.7150/jca.45378 (PMC7378928; doi:10.7150/jca.45378)
Supplement: Supplementary file 1 — Supplementary tables. [file jcav11p4933s1.pdf]

Table S1. The commonly up-regulated genes in four GSE datasets.

**Up-regulated genes**
COL4A2
CMTM3
PDPN
PLXDC2
IGFBP7
TEAD4
FGD6
PRRX1
TGM2
FBN1
PLAU
S100A10
APOE
COL3A1

DTL
BMP1
FLNA
CEBPB
LEF1
AFAP1L1
EDNRA
CDH11
GNB4
LGALS1
FZD2
GPR161
MSR1
AMIGO2
VCAN
HOXB7

SULF2
BAG2
LRP8
BUB1
COL5A1
TMEM158
RAB31
COL1A2
GREM1
NRP2
EHD2
ANTXR1
FAM83D
COL12A1
SPARC
CAP2

CST4
FN1
PMEPA1
PLPPR4
GPNMB
APOC1
FAM19A5
ADAM12
CLEC11A
OLFML2B
INHBA
RIN3
COL5A2
PDLIM7
SPHK1
SRPX2

THY1
COL6A3
RARRES1
CST2
TIMP1
COL8A1
MMP11
LY6E
MFAP2
COL1A1
NID2
CPXM1
SERPINH1
WISP1
BGN
HOXA10

CXCL8
DUXAP10
HOXC10
SPOCK1
ASPN
CRISPLD1
FKBP10
CLDN1
CTHRC1
ADAMTS2
CDH3
IGF2BP3
CEMIP
SPP1
SFRP4
SULF1

NOX4
CST1
THBS2
FAP
FNDC1
COL10A1
COL11A1

Table S2. The commonly down-regulated genes in four GSE datasets.

**Down-regulated genes**
GKN2
GKN1
ATP4A
ATP4B
AQP4
GIF

LIPF
KCNJ16
DPCR1
SOSTDC1
KCNE2
TRIM50
SCNN1G
CWH43
ESRRG
PGC
SLC28A2
PSAPL1
KRT20
VSIG1
LTF
CXCL17

AKR1B10
ALDOB
LOC643201
GSTA1
ADH7
CLIC6
CCKBR
CAPN9
MAL
SLC26A9
HRASLS2
MFSD4A
MUC5AC
FBP2
CHGA
CPA2

ALDH3A1
ADGRG2
VSTM2A
LINC00982
CAPN13
KIAA1324
CA9
TPCN2
PIK3C2G
SULT2A1
RDH12
SLC26A7
SSTR1
VSIG2
GATA6-AS1
HPGD

UPK1B
KCNJ15
ASCL1
DNER
SULT1C2
LINC00675
LINC01105
KAZALD1
SPINK2
TMED6
UGT2B15
ZBTB7C
SCNN1B
HAPLN1
AKR1C1
LYPD6B

ADTRP
IGH
CA2
RFX6
ACER2
CYP2C9
B3GNT6
PCAT18
GUCA2B
PKIB
SH3RF2
HHIP
AADAC
CYP2C18
RAB27B
MGAM

SPINK7
CNTN3
LINC01133
SULT1B1
CAPN8
ABCA8
SMIM6
AMPD1
JCHAIN
PBLD
ATP13A4
SMIM5
LRRC31
FOLR1
ENPP6
RNASE1

PLLP
NRG4
B4GALNT3
CYP2C19
SMIM24
FBXL13
LRRC66
LIFR
MYRF
RASSF6
ADAM28
FA2H
GATA5
SCIN
SGK2
TPH1

APOBEC1
ADRB2
LDHD
ACKR4
AKR7A3
OASL
SMPD3
XK
KLHDC7A
CYP3A5
TRPA1
ANG
FAM20A
TMPRSS2
DDX60
LOC400043

SLC16A7
ALDH1A1
KLF4
TRIQK
UBE2QL1
CYSTM1
SYTL2
METTL7A
GCNT2
EPN3
FER1L4
ITPKA
NKX2-3
FMO5
ASPA
PCBP1-AS1

MST1L
ARHGEF28
RAB27A
MAOA
KCNMB2
RPS6KA6
EPB41L4B
UBL3
NEDD4L
SELENBP1
SH3BGRL2
RBM47
PPID
NFE2L2
PPP1R36
PDGFD

GPRC5C
BCL2L14
SLC9A1
NTN4
CA4
THSD4
C1orf116
ALDH6A1
IQGAP2
TMEM171
HIPK2
WIPF3
MRAP2
DGKD
SLC41A2
KCNK10

TLR3
MYZAP
PXMP2
LOC202181
RNASE4
NOSTRIN
SCNN1A
CCL28
ELOVL6
NQO1
AKR1C3
SLC7A8
CYP3A43
DISP1
CD36
LOC101927391

TMEM116
DHRS7
ABCC5
PRDM16
OSBPL7
MAGI3
PELI2
TMEM220
PPFIBP2
MAGIX
GRAMD1C
RNF128
LOC101928881
TMEM92
TEX9
SPATA6

MAGI1

Table S3. The miRNA-mRNA pairs predicted by miRTarBase database.

**miRNA**	**mRNA**
hsa-miR-200c-3p	FN1
hsa-miR-200b-3p	FN1
hsa-miR-1-3p	FN1
hsa-let-7g-5p	FN1
hsa-miR-140-3p	FN1
hsa-miR-29c-3p	COL3A1
hsa-miR-29b-3p	COL3A1
hsa-miR-29a-3p	COL3A1
hsa-let-7b-5p	COL3A1
hsa-miR-767-5p	COL3A1
hsa-miR-29b-1-5p	COL3A1
hsa-miR-29c-3p	FBN1

hsa-miR-29a-3p	FBN1
hsa-miR-29b-3p	FBN1
hsa-miR-767-5p	FBN1
hsa-miR-486-5p	FBN1
hsa-miR-29a-3p	COL5A2
hsa-miR-29b-3p	COL5A2
hsa-miR-29c-3p	COL5A2
hsa-miR-767-5p	COL5A2
hsa-miR-27b-3p	THBS2
hsa-miR-135b-5p	THBS2
hsa-miR-29b-1-5p	COL5A1
hsa-miR-29c-3p	SPARC
hsa-miR-29a-3p	SPARC
hsa-miR-29b-3p	SPARC
hsa-miR-767-5p	SPARC
hsa-miR-211-5p	SPARC

hsa-miR-433-5p	SPARC
hsa-miR-1289	ATP4A

Table S4. The lncRNA-miRNA pairs predicted by miRNet database.

**miRNA**	**lncRNA**
hsa-mir-29a-3p	AC005154.5
hsa-mir-200b-3p	AC005154.5
hsa-mir-200c-3p	AC005154.5
hsa-mir-29a-3p	AC005154.6
hsa-mir-200b-3p	AC005154.6
hsa-mir-200c-3p	AC005154.6
hsa-mir-29a-3p	AC007036.5
hsa-mir-29a-3p	AC012146.7
hsa-mir-29a-3p	AP000304.2
hsa-mir-200b-3p	C11orf95
hsa-mir-200c-3p	C11orf95

hsa-mir-200b-3p	CTA-204B4.6
hsa-mir-200c-3p	CTA-204B4.6
hsa-mir-200b-3p	CTB-92J24.2
hsa-mir-200c-3p	CTB-92J24.2
hsa-mir-29a-3p	CTD-2116N17.1
hsa-mir-29a-3p	CTD-2339L15.1
hsa-mir-29a-3p	CTD-2517M14.5
hsa-mir-200b-3p	CTD-2630F21.1
hsa-mir-200c-3p	CTD-2630F21.1
hsa-mir-200b-3p	CTD-3099C6.9
hsa-mir-200c-3p	CTD-3099C6.9
hsa-mir-200b-3p	CTD-3185P2.1
hsa-mir-200c-3p	CTD-3185P2.1
hsa-mir-200b-3p	CWC15
hsa-mir-200c-3p	CWC15
hsa-mir-29a-3p	EMG1

hsa-mir-29a-3p	GAS5
hsa-mir-29a-3p	GS1-124K5.11
hsa-mir-29a-3p	H19
hsa-mir-29a-3p	HCP5
hsa-mir-29a-3p	HOXA-AS3
hsa-mir-29a-3p	HOXA-AS4
hsa-mir-29a-3p	KCNQ1OT1
hsa-mir-29a-3p	LIFR-AS1
hsa-mir-29a-3p	LINC00338
hsa-mir-29a-3p	LINC00511
hsa-mir-200b-3p	LINC00641
hsa-mir-200c-3p	LINC00641
hsa-mir-200b-3p	LINC00667
hsa-mir-200c-3p	LINC00667
hsa-mir-200b-3p	MALAT1
hsa-mir-200c-3p	MALAT1

hsa-mir-200b-3p	MAPKAPK5-AS1
hsa-mir-200c-3p	MAPKAPK5-AS1
hsa-mir-200b-3p	MATN1-AS1
hsa-mir-200c-3p	MATN1-AS1
hsa-mir-29a-3p	MIAT
hsa-mir-29a-3p	MLLT4-AS1
hsa-mir-29a-3p	OIP5-AS1
hsa-mir-200b-3p	OIP5-AS1
hsa-mir-200c-3p	OIP5-AS1
hsa-mir-200b-3p	PPP1R9B
hsa-mir-200c-3p	PPP1R9B
hsa-mir-200c-3p	RP11-1134I14.8
hsa-mir-29a-3p	RP11-145M9.4
hsa-mir-200b-3p	RP11-145M9.4
hsa-mir-200c-3p	RP11-145M9.4
hsa-mir-200b-3p	RP11-18F14.2

hsa-mir-200c-3p	RP11-18F14.2
hsa-mir-200b-3p	RP11-214C8.5
hsa-mir-200c-3p	RP11-214C8.5
hsa-mir-29a-3p	RP11-216F19.2
hsa-mir-29a-3p	RP11-220I1.1
hsa-mir-29a-3p	RP11-227G15.3
hsa-mir-200b-3p	RP11-277L2.2
hsa-mir-200c-3p	RP11-277L2.2
hsa-mir-29a-3p	RP11-280F2.2
hsa-mir-29a-3p	RP11-290F20.1
hsa-mir-29a-3p	RP11-303E16.8
hsa-mir-200b-3p	RP11-305E6.4
hsa-mir-200c-3p	RP11-305E6.4
hsa-mir-29a-3p	RP11-311C24.1
hsa-mir-29a-3p	RP11-347I19.3
hsa-mir-200b-3p	RP11-361F15.2

hsa-mir-200c-3p	RP11-361F15.2
hsa-mir-29a-3p	RP11-373L24.1
hsa-mir-200b-3p	RP11-379K17.4
hsa-mir-200c-3p	RP11-379K17.4
hsa-mir-200b-3p	RP11-403I13.4
hsa-mir-200c-3p	RP11-403I13.4
hsa-mir-29a-3p	RP11-429J17.2
hsa-mir-200b-3p	RP11-429J17.7
hsa-mir-200c-3p	RP11-429J17.7
hsa-mir-29a-3p	RP11-467L20.9
hsa-mir-200b-3p	RP11-473I1.10
hsa-mir-200c-3p	RP11-473I1.10
hsa-mir-29a-3p	RP11-480D4.3
hsa-mir-29a-3p	RP11-615I2.7
hsa-mir-29a-3p	RP11-618G20.1
hsa-mir-200b-3p	RP11-690D19.3

hsa-mir-200c-3p	RP11-690D19.3
hsa-mir-29a-3p	RP11-690G19.3
hsa-mir-200b-3p	RP11-91G21.2
hsa-mir-200c-3p	RP11-91G21.2
hsa-mir-200b-3p	RP13-507I23.1
hsa-mir-200c-3p	RP13-507I23.1
hsa-mir-29a-3p	RP4-665N4.8
hsa-mir-200b-3p	RP4-773N10.5
hsa-mir-200c-3p	RP4-773N10.5
hsa-mir-200b-3p	RP5-1085F17.3
hsa-mir-200c-3p	RP5-1085F17.3
hsa-mir-29a-3p	RP5-837J1.2
hsa-mir-200b-3p	RP6-24A23.7
hsa-mir-200c-3p	RP6-24A23.7
hsa-mir-200b-3p	SCAMP1
hsa-mir-200c-3p	SCAMP1

hsa-mir-29a-3p	SETD5-AS1
hsa-mir-29a-3p	SIK3-IT1
hsa-mir-29a-3p	SPPL2B
hsa-mir-29a-3p	TUG1
hsa-mir-29a-3p	U47924.19
hsa-mir-200b-3p	UBXN8
hsa-mir-200c-3p	UBXN8
hsa-mir-29a-3p	XIST
hsa-mir-200b-3p	XIST
hsa-mir-200c-3p	XIST
hsa-mir-29a-3p	ZNF518A
hsa-mir-200b-3p	ZNF518A
hsa-mir-200c-3p	ZNF518A
hsa-mir-29a-3p	ZNF761

Table S5. Sequences of primers using for qPCR.

ID	Forward Primer (5′-3′)	Reverse Primer (5′-3′)
miR-29a-3p	GCGTAGCACCATCTGAAAT	CAGTGCGTGTCGTGGAGT
U6	CGCAAGGATGACACG	GAGCAGGCTGGAGAA
H19	CCCACAACATGAAAGAAATGGTGC	CACCTTCGAGAGCCGATTCC
COL5A2	CCGGGTCTAGCTGGTGAAAG	TCTCCTCTAGGTCCTAACGGG
COL3A1	CGGCAATCCTGAACTTCCTG	ATCAGCTTCAGGGCCTTCTT
FBN1	GAGGCTGGGAACGTGAAG	AGTGCTGCTGTGATGCCG
SPARC	ATGACGACGGCACCTACAG	TCGCGTTGGGGTAACTTTTCA
GAPDH	AATGGACAACTGGTCGTGGAC	CCCTCCAGGGGATCTGTTTG

Table S6. GO and KEGG functional enrichment analysis.

ID	Term	P Value	Count	Associated Genes
GO:0043062	extracellular structure organization	4.1291E-28	34	ADAM12, ADAMTS2, ANTXR1, APOC1, APOE, BGN, BMP1, COL10A1, COL11A1, COL12A1, COL1A1, COL1A2, COL3A1, COL4A2, COL5A1, COL5A2, COL6A3, COL8A1, FAP, FBN1,

				FN1, GREM1, MFAP2, MMP11, NID2, OLFML2B, PDPN, SERPINH1, SPARC, SPP1, SULF1, SULF2, TIMP1, VCAN
GO:0030198	extracellular matrix organization	4.6022E-28	32	ADAM12, ADAMTS2, ANTXR1, BGN, BMP1, COL10A1, COL11A1, COL12A1, COL1A1, COL1A2, COL3A1, COL4A2, COL5A1, COL5A2, COL6A3, COL8A1, FAP, FBN1, FN1, GREM1, MFAP2, MMP11, NID2, OLFML2B, PDPN, SERPINH1, SPARC, SPP1, SULF1, SULF2, TIMP1, VCAN
GO:0030199	collagen fibril organization	7.1746E-15	11	ADAMTS2, COL11A1, COL12A1, COL1A1, COL1A2, COL3A1, COL5A1, COL5A2, GREM1, MMP11, SERPINH1
GO:0051216	cartilage development	1.201E-09	13	BMP1, CDH11, COL11A1, COL12A1, COL1A1, COL6A3, GREM1, INHBA, PRRX1, SERPINH1, SULF1, SULF2, TIMP1
GO:0061448	connective tissue development	2.8661E-09	14	BMP1, CDH11, COL11A1, COL12A1, COL1A1,COL5A1, COL6A3, GREM1, INHBA, PRRX1, SERPINH1, SULF1, SULF2, TIMP1
GO:0010810	regulation of cell-substrate adhesion	3.0566E-08	12	COL1A1, COL8A1, FLNA, FN1, GREM1, INHBA, LGALS1, PDPN, PLAU, S100A10, SPOCK1, THY1
GO:0060348	bone development	1.9105E-07	11	CDH11, COL12A1, COL1A1, COL6A3, FBN1, GREM1, SERPINH1, SFRP4, SPARC, SULF1, SULF2
GO:0034308	primary alcohol metabolic process	1.182E-07	10	ADH7, AKR1B10, AKR1C1, AKR1C3, ALDH1A1, RAB27A, RDH12, SULT1B1, SULT1C2, SULT2A1

GO:0006805	xenobiotic metabolic process	2.1874E-07	12	AADAC, AKR1C1, AKR7A3, ALDH3A1, CYP2C18, CYP2C19, CYP2C9, CYP3A5, GSTA1, NQO1, SULT1B1, UGT2B15
GO:0001676	long-chain fatty acid metabolic process	1.3839E-06	10	ADTRP, AKR1C1, AKR1C3, CYP2C18, CYP2C19, CYP2C9, ELOVL6, FA2H, GSTA1, HPGD
GO:0016614	oxidoreductase activity, acting on CH-OH group of donors	4.3311E-06	10	ADH7, AKR1B10, AKR1C1, AKR1C3, AKR7A3, ALDH3A1, HPGD, LDHD, LIPF, RDH12
GO:0071466	cellular response to xenobiotic stimulus	1.011E-05	12	AADAC, AKR1C1, AKR7A3, ALDH3A1, CYP2C18, CYP2C19, CYP2C9, CYP3A5, GSTA1, NQO1, SULT1B1, UGT2B15
GO:0071805	potassium ion transmembrane transport	7.8303E-05	11	ADRB2, ATP4A, ATP4B, KCNE2, KCNJ15, KCNJ16, KCNK10, KCNMB2, NEDD4L, SGK2, SLC9A1
GO:0016999	antibiotic metabolic process	6.8357E-05	10	ADH7, AKR1B10, AKR1C1, AKR1C3, ALDH1A1, LDHD, RNASE1, SULT1B1, SULT1C2, SULT2A1
GO:0044242	cellular lipid catabolic process	4.6583E-05	12	AADAC, ACER2, ADTRP, AKR1B10, AKR1C1, AKR1C3, ENPP6, FA2H, GSTA1, RAB27A, SMPD3, ZBTB7C
GO:0004497	monooxygenase activity	4.3377E-05	10	AKR1C1, AKR1C3, CYP2C18, CYP2C19, CYP2C9, CYP3A43, CYP3A5, FMO5, NOSTRIN, TPH1
GO:0015079	potassium ion transmembrane transporter activity	4.2156E-05	11	ADRB2, ATP4A, ATP4B, KCNE2, KCNJ15, KCNJ16, KCNK10, KCNMB2, NEDD4L, SGK2, SLC9A1
KEGG:04974	Protein digestion and absorption	1.0474E-10	10	COL10A1, COL11A1, COL12A1, COL1A1, COL1A2, COL3A1,

				COL4A2, COL5A1, COL5A2, COL6A3
KEGG:05200	Pathways in cancer	0.04064652	7	COL4A2, CXCL8, EDNRA, FN1, FZD2, GNB4, LEF1
KEGG:04151	PI3K-Akt signaling pathway	0.00121608	8	COL1A1, COL1A2, COL4A2, COL6A3, FN1, GNB4, SPP1, THBS2
KEGG:04510	Focal adhesion	2.3157E-05	8	COL1A1, COL1A2, COL4A2, COL6A3, FLNA, FN1, SPP1, THBS2
KEGG:04512	ECM-receptor interaction	5.4102E-07	7	COL1A1, COL1A2, COL4A2, COL6A3, FN1, SPP1, THBS2
KEGG:04926	Relaxin signaling pathway	0.00111533	5	COL1A1, COL1A2, COL3A1, COL4A2, GNB4
KEGG:04933	AGE-RAGE signaling pathway in diabetic complications	2.098E-06	7	COL1A1, COL1A2, COL3A1, COL4A2, CXCL8, FN1, NOX4
KEGG:05146	Amoebiasis	2.3082E-05	6	COL1A1, COL1A2, COL3A1, COL4A2, CXCL8, FN1
KEGG:05165	Human papillomavirus infection	0.00076979	8	COL1A1, COL1A2, COL4A2, COL6A3, FN1, FZD2, SPP1, THBS2
KEGG:04971	Gastric acid secretion	4.939E-08	10	ATP4A, ATP4B, CA2, CCKBR, KCNE2, KCNJ15, KCNJ16, KCNK10, SLC26A7, SLC9A1
KEGG:05204	Chemical carcinogenesis	1.1793E-07	10	ADH7, ALDH3A1, CYP2C18, CYP2C19, CYP2C9, CYP3A43, CYP3A5, GSTA1, SULT2A1, UGT2B15
KEGG:00982	Drug metabolism	4.2285E-07	9	ADH7, ALDH3A1, CYP2C19, CYP2C9, CYP3A5, FMO5, GSTA1, MAOA, UGT2B15
KEGG:00980	Metabolism of xenobiotics by cytochrome P450	6.7706E-07	9	ADH7, AKR1C1, AKR7A3, ALDH3A1, CYP2C9, CYP3A5, GSTA1, SULT2A1, UGT2B15

KEGG:00830	Retinol metabolism	2.8949E-05	7	ADH7, ALDH1A1, CYP2C18, CYP2C9, CYP3A5, RDH12, UGT2B15
KEGG:04960	Aldosterone-regulated sodium reabsorption	0.00144629	4	NEDD4L, SCNN1A, SCNN1B, SCNN1G
KEGG:00910	Nitrogen metabolism	0.00139889	3	CA2, CA4, CA9
KEGG:00340	Histidine metabolism	0.00343639	3	ALDH3A1, ASPA, MAOA
